# *N* ^1^,*N* ^1^-Dimethyl­propane-1,2-diaminium bis­(6-carb­oxy­pyridine-2-carboxyl­ate) monohydrate

**DOI:** 10.1107/S1600536811009287

**Published:** 2011-03-19

**Authors:** Hossein Aghabozorg, Mahsa Foroughian, Alireza Foroumadi, Giuseppe Bruno, Hadi Amiri Rudbari

**Affiliations:** a Faculty of Chemistry, Islamic Azad University, North Tehran Branch, Tehran, Iran; b Drug Design and Development Research Center, Tehran University of Medical Sciences, Tehran, Iran; c Dipartimento di Chimica Inorganica, Vill. S. Agata, Salita Sperone 31, Universita di Messina, 98166 Messina, Italy

## Abstract

The asymmetric unit of the title proton-transfer compound, C_5_H_16_N_2_^2+^·2C_7_H_4_NO_4_^−^·H_2_O, consists of two mono-deproton­ated pyridine-2,6-dicarb­oxy­lic acid mol­ecules as anions, *viz.* (py-2,6-dcH)^−^, one diprotonated *N* ^1^,*N* ^1^-dimethyl­propane-1,2-diamine mol­ecule as a cation, *viz.* (dmpdaH_2_)^2+^, and one water mol­ecule. The crystal packing shows extensive O—H⋯O, N—H⋯O, N—H⋯N and O—H⋯N and weak inter­molecular C—H⋯O hydrogen bonds. These inter­actions link the (dmpdaH_2_)^2+^ cation, the (py-2,6-dcH)^−^ anions and water mol­ecule and play an important role in the stabilization of crystal packing.

## Related literature

For background to proton-transfer compounds, see: Aghabozorg *et al.* (2008 ▶). For related structures, see: Aghabozorg, Bayan *et al.* (2011 ▶); Aghabozorg, Mofidi Rouchi *et al.* (2011 ▶); Aghabozorg, Saemi *et al.* (2011 ▶); Sharif *et al.* (2010 ▶).
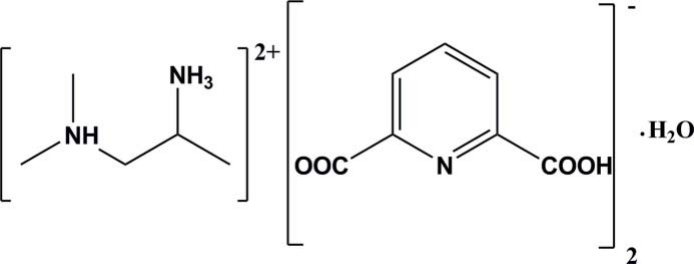


## Experimental

### 

#### Crystal data

C_5_H_16_N_2_^2+^·2C_7_H_4_NO_4_^−^·H_2_O
*M* *_r_* = 454.44
Orthorhombic, 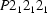

*a* = 11.826 (8) Å
*b* = 13.376 (8) Å
*c* = 13.479 (8) Å
*V* = 2132 (2) Å^3^
*Z* = 4
Mo *K*α radiation
μ = 0.11 mm^−1^
*T* = 293 K
0.42 × 0.38 × 0.22 mm

#### Data collection

Bruker APEXII CCD diffractometer
Absorption correction: multi-scan (*SADABS*; Bruker, 2008 ▶) *T*_min_ = 0.708, *T*_max_ = 0.746
70173 measured reflections
4650 independent reflections
4509 reflections with *I* > 2σ(*I*)
*R*_int_ = 0.025

#### Refinement

*R*[*F* ^2^ > 2σ(*F* ^2^)] = 0.029
*wR*(*F* ^2^) = 0.084
*S* = 1.07
4650 reflections
298 parameters
3 restraints
H atoms treated by a mixture of independent and constrained refinement
Δρ_max_ = 0.23 e Å^−3^
Δρ_min_ = −0.18 e Å^−3^

### 

Data collection: *APEX2* (Bruker, 2008 ▶); cell refinement: *SAINT* (Bruker, 2008 ▶); data reduction: *SAINT*; program(s) used to solve structure: *SHELXS97* (Sheldrick, 2008 ▶); program(s) used to refine structure: *SHELXL97* (Sheldrick, 2008 ▶); molecular graphics: *SHELXTL* (Sheldrick, 2008 ▶); software used to prepare material for publication: *SHELXTL*.

## Supplementary Material

Crystal structure: contains datablocks I, global. DOI: 10.1107/S1600536811009287/vm2081sup1.cif

Structure factors: contains datablocks I. DOI: 10.1107/S1600536811009287/vm2081Isup2.hkl

Additional supplementary materials:  crystallographic information; 3D view; checkCIF report

## Figures and Tables

**Table 1:** Hydrogen-bond geometry (Å, °)

*D*—H⋯*A*	*D*—H	H⋯*A*	*D*⋯*A*	*D*—H⋯*A*
N1—H1⋯O9	0.91	1.82	2.711 (2)	165
N2—H1*A*⋯O9	0.89	2.55	3.392 (3)	158
N2—H1*A*⋯N3	0.89	2.40	2.988 (2)	124
N2—H2*A*⋯O5^i^	0.89	2.25	2.948 (2)	135
N2—H2*A*⋯N4^i^	0.89	2.32	3.123 (3)	151
O3—H3⋯O5	0.82	1.68	2.473 (2)	162
N2—H3*A*⋯O2^ii^	0.89	2.05	2.845 (2)	148
N2—H3*A*⋯O7^i^	0.89	2.30	2.902 (2)	125
O7—H7⋯O1^iii^	0.82	1.75	2.535 (2)	159
O9—H9*A*⋯O1	0.80 (2)	2.30 (2)	2.900 (2)	132 (2)
O9—H9*A*⋯N3	0.80 (2)	2.38 (2)	3.107 (3)	152 (2)
O9—H9*B*⋯O3	0.83 (2)	2.33 (3)	2.958 (3)	133 (3)
O9—H9*B*⋯O6	0.83 (2)	2.16 (2)	2.844 (2)	139 (3)
C3—H3*B*⋯O6^iv^	0.97	2.58	3.541 (3)	172
C3—H3*C*⋯O2^ii^	0.97	2.20	3.057 (3)	147
C4—H4⋯O4^i^	0.98	2.45	3.349 (3)	152
C15—H15⋯O8^v^	0.93	2.48	3.166 (3)	131
C2—H20*A*⋯O4^i^	0.96	2.57	3.443 (3)	152

Symmetry codes: (i) 
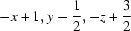
; (ii) 
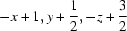
; (iii) 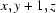; (iv) 
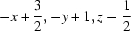
; (v) 
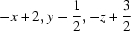
.

**Fig. 1.**
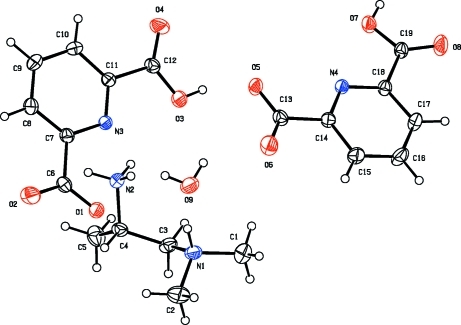
The molecular structure of the title compound. Displacement ellipsoids are drawn at 30% probability level.

**Fig. 2.**
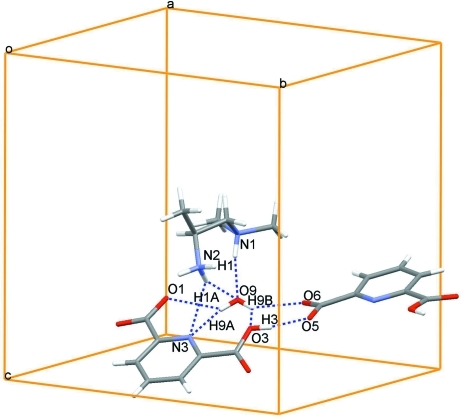
The packing diagram of the title compound showing hydrogen bonding interactions in the asymmetric unit as blue dashed lines.

**Fig. 3.**
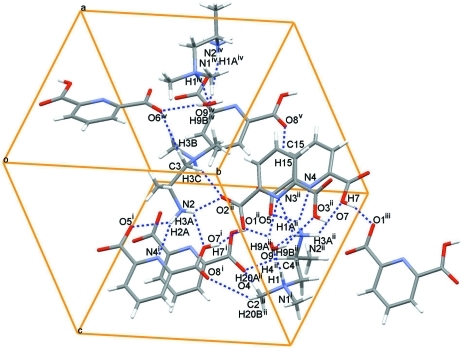
The packing diagram of the title compound showing all hydrogen bonding interactions as blue dashed lines. Symmetry codes: (i) -x+1, y-1/2, -z+3/2; (ii) -x+1, y+1/2, -z+3/2; (iii) x, y+1, z; (iv) -x+3/2, -y+1, z-1/2; (v) -x+2, y-1/2, -z+3/2.

## supplementary crystallographic information

### Comment

Proton transfer is very important in chemistry, biochemistry and medicinal chemistry. In order to synthesize new types of proton transfer compounds our research group focusses on forming new compounds from pyridine dicarboxcylic acids (Aghabozorg *et al.* 2008) and different organic bases with nitrogen donor sites such as propane-1,3-diamine (Aghabozorg, Bayan *et al.*, 2011), diethylenetriamine (Aghabozorg, Saemi *et al.*, 2011) and 2-amino-4-methylpyridine (Aghabozorg, Mofidi Rouchi *et al.*, 2011; Sharif *et al.*, 2010).

The asymmetric unit of the title compound consists of two mono deprotonated pyridine-2,6-dicarboxylic acid as anion, one diprotonated *N*,*N*'-dimethyl-1,2-propanediamine as cation, and one water molecule (Fig. 1). In the crystal packing there are extensive O—H···O, N—H···O, N—H···N, O—H···N and weak intermolecular C—H···O hydrogen bonds (Table 1, Fig. 2, Fig. 3). The *N*,*N*'-dimethyl-1,2-propanediaminium (dmpdaH_2_)^2+^ cation is linked to the water molecule by N—H···O hydrogen bonds. This water molecule also connects the cationic part to the anionic part by N—H···O, O—H···N and O—H···O hydrogen bonds. These hydrogen bonds play an important role in the stabilization of the crystal packing.

### Experimental

The solution of pyridine-2,6-dicarboxylic acid (0.334 g, 2 mmol) in 15 ml water was reacted with a solution of *N*,*N*'-dimethyl-1,2-propanediamine (0.258 ml, 2 mmol) in 7 ml water in 1:1 molar ratios. The reaction mixture was stirred for 2hrs at 298 K. The colorless crystals of the title compound appeared after slow evaporation of solvent at room temperature in darkness (m.p:177°C).

### Refinement

The hydrogen atoms of the water molecule were found in difference Fourier map and refined isotropically with distance restraints of O—H 0.80 (2) and 0.83 (2) for H9A and H9B, respectively and a H···H distance of 1.276 (4). The other H-atoms were included at calculated positions and treated as riding atoms: O—H = 0.82 Å, N—H = 0.91, 0.89 Å for NH and NH_3_, C—H = 0.93, 0.98, 0.97 and 0.96 Å for CH(aliphatic), CH(aromatic), CH_2_ and CH_3_ hydrogen atoms, respectively. These H-atoms were refined with *Uiso*(H) = *k* × *Ueq*(parent atom), where *k* = 1.5 for OH and CH_3_ H-atoms, and *k* = 1.2 for all other H-atoms.

### Crystal data

**Table:**

C_5_H_16_N_2_^2+^·2C_7_H_4_NO_4_^−^·H_2_O	*F*(000) = 960
*M**_r_* = 454.44	*D*_x_ = 1.416 Mg m^−^^3^
Orthorhombic, *P*2_1_2_1_2_1_	Mo *K*α radiation, λ = 0.71073 Å
Hall symbol: P 2ac 2ab	Cell parameters from 296(2) reflections
*a* = 11.826 (8) Å	θ = 2.2–27.0°
*b* = 13.376 (8) Å	µ = 0.11 mm^−^^1^
*c* = 13.479 (8) Å	*T* = 293 K
*V* = 2132 (2) Å^3^	Irregular, colorless
*Z* = 4	0.42 × 0.38 × 0.22 mm

### Data collection

**Table:**

Bruker APEXII CCD diffractometer	4650 independent reflections
Radiation source: fine-focus sealed tube	4509 reflections with *I* > 2σ(*I*)
graphite	*R*_int_ = 0.025
φ and ω scans	θ_max_ = 27.0°, θ_min_ = 2.2°
Absorption correction: multi-scan (*SADABS*; Bruker, 2008)	*h* = −15→15
*T*_min_ = 0.708, *T*_max_ = 0.746	*k* = −17→17
70173 measured reflections	*l* = −17→17

### Refinement

**Table:**

Refinement on *F*^2^	Secondary atom site location: difference Fourier map
Least-squares matrix: full	Hydrogen site location: inferred from neighbouring sites
*R*[*F*^2^ > 2σ(*F*^2^)] = 0.029	H atoms treated by a mixture of independent and constrained refinement
*wR*(*F*^2^) = 0.084	*w* = 1/[σ^2^(*F*_o_^2^) + (0.0516*P*)^2^ + 0.2616*P*] where *P* = (*F*_o_^2^ + 2*F*_c_^2^)/3
*S* = 1.07	(Δ/σ)_max_ = 0.001
4650 reflections	Δρ_max_ = 0.23 e Å^−^^3^
298 parameters	Δρ_min_ = −0.18 e Å^−^^3^
3 restraints	Extinction correction: *SHELXL97* (Sheldrick, 2008), Fc^*^=kFc[1+0.001xFc^2^λ^3^/sin(2θ)]^-1/4^
Primary atom site location: structure-invariant direct methods	Extinction coefficient: 0.0064 (13)

### Special details

**Table:**

Geometry. All s.u.'s (except the s.u. in the dihedral angle between two l.s. planes) are estimated using the full covariance matrix. The cell s.u.'s are taken into account individually in the estimation of s.u.'s in distances, angles and torsion angles; correlations between s.u.'s in cell parameters are only used when they are defined by crystal symmetry. An approximate (isotropic) treatment of cell s.u.'s is used for estimating s.u.'s involving l.s. planes.
Refinement. Refinement of *F*^2^ against ALL reflections. The weighted *R*-factor *wR* and goodness of fit *S* are based on *F*^2^, conventional *R*-factors *R* are based on *F*, with *F* set to zero for negative *F*^2^. The threshold expression of *F*^2^ > 2σ(*F*^2^) is used only for calculating *R*-factors(gt) *etc*. and is not relevant to the choice of reflections for refinement. *R*-factors based on *F*^2^ are statistically about twice as large as those based on *F*, and *R*- factors based on ALL data will be even larger.

### Fractional atomic coordinates and isotropic or equivalent isotropic displacement parameters (Å^2^)

**Table:**

	*x*	*y*	*z*	*U*_iso_*/*U*_eq_	
C1	0.82378 (16)	0.49387 (15)	0.59189 (15)	0.0608 (4)	
H10A	0.7917	0.5578	0.6078	0.091*	
H10B	0.8457	0.4929	0.5233	0.091*	
H10C	0.8890	0.4823	0.6328	0.091*	
C2	0.78465 (16)	0.31373 (12)	0.58604 (14)	0.0589 (4)	
H20A	0.7278	0.2639	0.5979	0.088*	
H20B	0.8493	0.3005	0.6271	0.088*	
H20C	0.8067	0.3119	0.5175	0.088*	
C3	0.63379 (12)	0.43601 (10)	0.55191 (10)	0.0410 (3)	
H3B	0.6481	0.4194	0.4830	0.049*	
H3C	0.6194	0.5073	0.5552	0.049*	
C4	0.52821 (12)	0.38176 (9)	0.58465 (9)	0.0352 (3)	
H4	0.5460	0.3112	0.5963	0.042*	
C5	0.44060 (16)	0.38990 (14)	0.50382 (12)	0.0557 (4)	
H5A	0.3731	0.3555	0.5239	0.084*	
H5B	0.4694	0.3603	0.4440	0.084*	
H5C	0.4235	0.4591	0.4921	0.084*	
C6	0.51400 (11)	0.21400 (9)	0.86340 (10)	0.0361 (3)	
C7	0.44510 (11)	0.29707 (9)	0.91060 (9)	0.0323 (2)	
C8	0.34944 (14)	0.27536 (10)	0.96579 (11)	0.0469 (3)	
H8	0.3263	0.2095	0.9746	0.056*	
C9	0.28930 (15)	0.35256 (12)	1.00737 (13)	0.0547 (4)	
H9	0.2244	0.3398	1.0442	0.066*	
C10	0.32657 (12)	0.44927 (11)	0.99364 (11)	0.0433 (3)	
H10	0.2881	0.5029	1.0218	0.052*	
C11	0.42230 (10)	0.46459 (9)	0.93714 (9)	0.0309 (2)	
C12	0.46436 (11)	0.56979 (9)	0.91995 (9)	0.0347 (3)	
C13	0.74414 (11)	0.71223 (9)	0.80029 (10)	0.0360 (3)	
C14	0.81762 (10)	0.79792 (9)	0.76580 (10)	0.0327 (2)	
C15	0.92660 (12)	0.77789 (11)	0.73243 (12)	0.0446 (3)	
H15	0.9531	0.7125	0.7291	0.054*	
C16	0.99431 (12)	0.85656 (12)	0.70449 (13)	0.0490 (4)	
H16	1.0672	0.8452	0.6812	0.059*	
C17	0.95259 (11)	0.95225 (11)	0.71152 (10)	0.0402 (3)	
H17	0.9975	1.0069	0.6951	0.048*	
C18	0.84204 (10)	0.96563 (9)	0.74366 (9)	0.0315 (2)	
C19	0.79612 (10)	1.07016 (9)	0.75010 (9)	0.0330 (2)	
N1	0.73847 (10)	0.41412 (9)	0.61018 (8)	0.0399 (3)	
H1	0.7203	0.4150	0.6758	0.048*	
N2	0.47949 (9)	0.42609 (8)	0.67685 (8)	0.0340 (2)	
H1A	0.5298	0.4217	0.7258	0.051*	
H2A	0.4171	0.3930	0.6936	0.051*	
H3A	0.4626	0.4900	0.6661	0.051*	
N3	0.48126 (8)	0.39046 (7)	0.89568 (7)	0.0291 (2)	
N4	0.77430 (8)	0.89011 (7)	0.76994 (7)	0.0295 (2)	
O1	0.59431 (8)	0.23905 (6)	0.80781 (8)	0.0420 (2)	
O2	0.48705 (13)	0.12753 (7)	0.88430 (10)	0.0636 (4)	
O3	0.54684 (9)	0.57393 (7)	0.85809 (8)	0.0454 (2)	
H3	0.5668	0.6322	0.8511	0.068*	
O4	0.42263 (12)	0.63994 (8)	0.96310 (10)	0.0592 (3)	
O5	0.64280 (8)	0.73404 (6)	0.82184 (8)	0.0417 (2)	
O6	0.78683 (10)	0.62868 (7)	0.80691 (11)	0.0570 (3)	
O7	0.68767 (8)	1.07388 (7)	0.76530 (8)	0.0428 (2)	
H7	0.6673	1.1324	0.7681	0.064*	
O8	0.85621 (10)	1.14190 (8)	0.74214 (11)	0.0578 (3)	
O9	0.72243 (10)	0.42389 (9)	0.81056 (8)	0.0489 (3)	
H9A	0.6703 (19)	0.3950 (18)	0.835 (2)	0.099 (9)*	
H9B	0.711 (3)	0.4830 (14)	0.827 (2)	0.115 (11)*	

### Atomic displacement parameters (Å^2^)

**Table:**

	*U*^11^	*U*^22^	*U*^33^	*U*^12^	*U*^13^	*U*^23^
C1	0.0545 (9)	0.0633 (10)	0.0645 (10)	−0.0123 (8)	0.0124 (8)	−0.0003 (8)
C2	0.0586 (10)	0.0474 (9)	0.0706 (10)	0.0237 (8)	0.0006 (8)	−0.0045 (8)
C3	0.0424 (7)	0.0358 (6)	0.0446 (7)	0.0094 (6)	0.0129 (5)	0.0066 (6)
C4	0.0431 (7)	0.0256 (5)	0.0370 (6)	0.0036 (5)	0.0063 (5)	−0.0008 (5)
C5	0.0664 (10)	0.0583 (9)	0.0423 (7)	−0.0145 (8)	−0.0085 (7)	−0.0005 (7)
C6	0.0419 (6)	0.0232 (5)	0.0431 (6)	0.0004 (5)	0.0016 (5)	0.0027 (5)
C7	0.0382 (6)	0.0248 (5)	0.0340 (5)	−0.0014 (5)	0.0030 (5)	0.0020 (4)
C8	0.0547 (8)	0.0329 (7)	0.0529 (8)	−0.0130 (6)	0.0179 (7)	−0.0007 (6)
C9	0.0528 (9)	0.0525 (8)	0.0587 (9)	−0.0150 (7)	0.0298 (8)	−0.0087 (7)
C10	0.0406 (7)	0.0406 (7)	0.0489 (7)	0.0015 (5)	0.0156 (6)	−0.0094 (6)
C11	0.0322 (6)	0.0276 (5)	0.0327 (5)	0.0004 (5)	0.0011 (5)	−0.0007 (4)
C12	0.0375 (6)	0.0256 (5)	0.0411 (6)	0.0034 (5)	−0.0008 (5)	−0.0004 (5)
C13	0.0369 (6)	0.0256 (5)	0.0455 (7)	0.0014 (5)	−0.0014 (5)	−0.0007 (5)
C14	0.0296 (6)	0.0273 (5)	0.0412 (6)	0.0026 (4)	0.0001 (5)	0.0010 (5)
C15	0.0341 (6)	0.0351 (6)	0.0647 (9)	0.0091 (5)	0.0054 (6)	0.0012 (6)
C16	0.0283 (6)	0.0533 (8)	0.0654 (9)	0.0069 (6)	0.0112 (6)	0.0066 (7)
C17	0.0292 (6)	0.0420 (7)	0.0495 (7)	−0.0043 (5)	0.0044 (5)	0.0083 (6)
C18	0.0294 (5)	0.0295 (6)	0.0357 (6)	−0.0009 (4)	−0.0008 (5)	0.0046 (4)
C19	0.0317 (5)	0.0283 (5)	0.0391 (6)	−0.0024 (5)	0.0002 (5)	0.0053 (5)
N1	0.0420 (6)	0.0376 (6)	0.0400 (5)	0.0045 (5)	0.0107 (5)	−0.0022 (4)
N2	0.0305 (5)	0.0362 (5)	0.0353 (5)	−0.0017 (4)	0.0039 (4)	−0.0012 (4)
N3	0.0299 (5)	0.0238 (4)	0.0335 (5)	0.0005 (4)	0.0034 (4)	0.0018 (4)
N4	0.0267 (4)	0.0255 (4)	0.0363 (5)	0.0006 (4)	0.0008 (4)	0.0017 (4)
O1	0.0368 (4)	0.0260 (4)	0.0632 (6)	0.0017 (4)	0.0110 (4)	−0.0009 (4)
O2	0.0880 (9)	0.0216 (4)	0.0814 (8)	0.0001 (5)	0.0362 (7)	0.0061 (5)
O3	0.0495 (5)	0.0229 (4)	0.0637 (6)	−0.0027 (4)	0.0157 (5)	0.0024 (4)
O4	0.0746 (8)	0.0288 (5)	0.0742 (8)	0.0032 (5)	0.0236 (7)	−0.0083 (5)
O5	0.0334 (4)	0.0256 (4)	0.0660 (6)	−0.0011 (4)	0.0090 (4)	0.0041 (4)
O6	0.0486 (6)	0.0267 (5)	0.0956 (9)	0.0067 (4)	0.0086 (6)	0.0069 (5)
O7	0.0325 (4)	0.0244 (4)	0.0714 (6)	0.0015 (3)	0.0023 (4)	−0.0022 (4)
O8	0.0448 (6)	0.0313 (5)	0.0973 (9)	−0.0083 (4)	0.0141 (6)	0.0070 (5)
O9	0.0454 (6)	0.0497 (6)	0.0516 (6)	0.0039 (5)	0.0127 (5)	−0.0008 (5)

### Geometric parameters (Å, °)

**Table:**

C1—N1	1.489 (2)	C10—H10	0.9300
C1—H10A	0.9600	C11—N3	1.3347 (15)
C1—H10B	0.9600	C11—C12	1.5104 (16)
C1—H10C	0.9600	C12—O4	1.2094 (16)
C2—N1	1.4857 (18)	C12—O3	1.2843 (16)
C2—H20A	0.9600	C13—O6	1.2296 (16)
C2—H20B	0.9600	C13—O5	1.2672 (16)
C2—H20C	0.9600	C13—C14	1.5117 (17)
C3—N1	1.4950 (19)	C14—N4	1.3364 (15)
C3—C4	1.5101 (18)	C14—C15	1.3910 (18)
C3—H3B	0.9700	C15—C16	1.375 (2)
C3—H3C	0.9700	C15—H15	0.9300
C4—N2	1.4926 (15)	C16—C17	1.375 (2)
C4—C5	1.507 (2)	C16—H16	0.9300
C4—H4	0.9800	C17—C18	1.3889 (17)
C5—H5A	0.9600	C17—H17	0.9300
C5—H5B	0.9600	C18—N4	1.3371 (15)
C5—H5C	0.9600	C18—C19	1.5025 (16)
C6—O2	1.2323 (16)	C19—O8	1.1988 (16)
C6—O1	1.2553 (16)	C19—O7	1.2996 (15)
C6—C7	1.5177 (17)	N1—H1	0.9100
C7—N3	1.3356 (15)	N2—H1A	0.8900
C7—C8	1.3848 (18)	N2—H2A	0.8900
C8—C9	1.373 (2)	N2—H3A	0.8900
C8—H8	0.9300	O3—H3	0.8200
C9—C10	1.379 (2)	O7—H7	0.8200
C9—H9	0.9300	O9—H9A	0.799 (17)
C10—C11	1.3798 (18)	O9—H9B	0.830 (17)
			
N1—C1—H10A	109.5	N3—C11—C10	123.33 (11)
N1—C1—H10B	109.5	N3—C11—C12	117.12 (10)
H10A—C1—H10B	109.5	C10—C11—C12	119.55 (11)
N1—C1—H10C	109.5	O4—C12—O3	126.07 (13)
H10A—C1—H10C	109.5	O4—C12—C11	120.98 (12)
H10B—C1—H10C	109.5	O3—C12—C11	112.94 (10)
N1—C2—H20A	109.5	O6—C13—O5	125.52 (13)
N1—C2—H20B	109.5	O6—C13—C14	118.39 (12)
H20A—C2—H20B	109.5	O5—C13—C14	116.08 (11)
N1—C2—H20C	109.5	N4—C14—C15	123.12 (12)
H20A—C2—H20C	109.5	N4—C14—C13	117.80 (11)
H20B—C2—H20C	109.5	C15—C14—C13	119.08 (11)
N1—C3—C4	115.91 (11)	C16—C15—C14	118.73 (13)
N1—C3—H3B	108.3	C16—C15—H15	120.6
C4—C3—H3B	108.3	C14—C15—H15	120.6
N1—C3—H3C	108.3	C15—C16—C17	118.99 (13)
C4—C3—H3C	108.3	C15—C16—H16	120.5
H3B—C3—H3C	107.4	C17—C16—H16	120.5
N2—C4—C5	107.93 (12)	C16—C17—C18	118.63 (12)
N2—C4—C3	111.82 (11)	C16—C17—H17	120.7
C5—C4—C3	108.80 (11)	C18—C17—H17	120.7
N2—C4—H4	109.4	N4—C18—C17	123.32 (12)
C5—C4—H4	109.4	N4—C18—C19	118.11 (10)
C3—C4—H4	109.4	C17—C18—C19	118.57 (11)
C4—C5—H5A	109.5	O8—C19—O7	124.64 (12)
C4—C5—H5B	109.5	O8—C19—C18	121.69 (11)
H5A—C5—H5B	109.5	O7—C19—C18	113.67 (10)
C4—C5—H5C	109.5	C2—N1—C1	111.23 (13)
H5A—C5—H5C	109.5	C2—N1—C3	111.49 (12)
H5B—C5—H5C	109.5	C1—N1—C3	109.50 (12)
O2—C6—O1	125.64 (13)	C2—N1—H1	108.2
O2—C6—C7	116.91 (12)	C1—N1—H1	108.2
O1—C6—C7	117.45 (10)	C3—N1—H1	108.2
N3—C7—C8	122.59 (12)	C4—N2—H1A	109.5
N3—C7—C6	116.73 (11)	C4—N2—H2A	109.5
C8—C7—C6	120.68 (11)	H1A—N2—H2A	109.5
C9—C8—C7	118.99 (12)	C4—N2—H3A	109.5
C9—C8—H8	120.5	H1A—N2—H3A	109.5
C7—C8—H8	120.5	H2A—N2—H3A	109.5
C8—C9—C10	119.01 (13)	C11—N3—C7	117.68 (10)
C8—C9—H9	120.5	C14—N4—C18	117.15 (10)
C10—C9—H9	120.5	C12—O3—H3	109.5
C9—C10—C11	118.40 (12)	C19—O7—H7	109.5
C9—C10—H10	120.8	H9A—O9—H9B	103 (3)
C11—C10—H10	120.8		
			
N1—C3—C4—N2	75.97 (14)	N4—C14—C15—C16	1.6 (2)
N1—C3—C4—C5	−164.91 (12)	C13—C14—C15—C16	−177.73 (14)
O2—C6—C7—N3	173.61 (14)	C14—C15—C16—C17	0.7 (2)
O1—C6—C7—N3	−5.34 (18)	C15—C16—C17—C18	−2.0 (2)
O2—C6—C7—C8	−6.7 (2)	C16—C17—C18—N4	1.2 (2)
O1—C6—C7—C8	174.39 (14)	C16—C17—C18—C19	−179.26 (13)
N3—C7—C8—C9	−0.3 (2)	N4—C18—C19—O8	168.74 (14)
C6—C7—C8—C9	179.99 (14)	C17—C18—C19—O8	−10.81 (19)
C7—C8—C9—C10	−0.6 (3)	N4—C18—C19—O7	−10.92 (16)
C8—C9—C10—C11	1.0 (3)	C17—C18—C19—O7	169.53 (12)
C9—C10—C11—N3	−0.5 (2)	C4—C3—N1—C2	74.31 (15)
C9—C10—C11—C12	179.35 (14)	C4—C3—N1—C1	−162.17 (12)
N3—C11—C12—O4	−172.38 (13)	C10—C11—N3—C7	−0.30 (18)
C10—C11—C12—O4	7.7 (2)	C12—C11—N3—C7	179.80 (11)
N3—C11—C12—O3	6.62 (16)	C8—C7—N3—C11	0.73 (19)
C10—C11—C12—O3	−173.29 (12)	C6—C7—N3—C11	−179.55 (11)
O6—C13—C14—N4	−172.34 (13)	C15—C14—N4—C18	−2.43 (19)
O5—C13—C14—N4	6.38 (18)	C13—C14—N4—C18	176.91 (11)
O6—C13—C14—C15	7.0 (2)	C17—C18—N4—C14	1.00 (18)
O5—C13—C14—C15	−174.25 (13)	C19—C18—N4—C14	−178.52 (11)

### Hydrogen-bond geometry (Å, °)

**Table:**

*D*—H···*A*	*D*—H	H···*A*	*D*···*A*	*D*—H···*A*
N1—H1···O9	0.91	1.82	2.711 (2)	165
N2—H1A···O9	0.89	2.55	3.392 (3)	158
N2—H1A···N3	0.89	2.40	2.988 (2)	124
N2—H2A···O5^i^	0.89	2.25	2.948 (2)	135
N2—H2A···N4^i^	0.89	2.32	3.123 (3)	151
O3—H3···O5	0.82	1.68	2.473 (2)	162
N2—H3A···O2^ii^	0.89	2.05	2.845 (2)	148
N2—H3A···O7^i^	0.89	2.30	2.902 (2)	125
O7—H7···O1^iii^	0.82	1.75	2.535 (2)	159
O9—H9A···O1	0.80 (2)	2.30 (2)	2.900 (2)	132 (2)
O9—H9A···N3	0.80 (2)	2.38 (2)	3.107 (3)	152 (2)
O9—H9B···O3	0.83 (2)	2.33 (3)	2.958 (3)	133 (3)
O9—H9B···O6	0.83 (2)	2.16 (2)	2.844 (2)	139 (3)
C3—H3B···O6^iv^	0.97	2.58	3.541 (3)	172
C3—H3C···O2^ii^	0.97	2.20	3.057 (3)	147
C4—H4···O4^i^	0.98	2.45	3.349 (3)	152
C15—H15···O8^v^	0.93	2.48	3.166 (3)	131
C2—H20A···O4^i^	0.96	2.57	3.443 (3)	152

Symmetry codes: (i) −*x*+1, *y*−1/2, −*z*+3/2; (ii) −*x*+1, *y*+1/2, −*z*+3/2; (iii) *x*, *y*+1, *z*; (iv) −*x*+3/2, −*y*+1, *z*−1/2; (v) −*x*+2, *y*−1/2, −*z*+3/2.
